# NET Formation in Bullous Pemphigoid Patients With Relapse Is Modulated by IL-17 and IL-23 Interplay

**DOI:** 10.3389/fimmu.2019.00701

**Published:** 2019-04-04

**Authors:** Delphine Giusti, Estela Bini, Christine Terryn, Kevin Didier, Sébastien Le Jan, Grégory Gatouillat, Anne Durlach, Stéphane Nesmond, Celine Muller, Philippe Bernard, Frank Antonicelli, Bach Nga Pham

**Affiliations:** ^1^Laboratory of Dermatology, Faculty of Medicine of Reims, University of Champagne-Ardenne, Reims, France; ^2^Laboratory of Immunology, Reims University Hospital, University of Champagne-Ardenne, Reims, France; ^3^PICT Platform, University of Reims Champagne-Ardenne, Reims, France; ^4^Laboratory of Pathology, Reims University Hospital, Reims, France; ^5^Department of Dermatology, Reims University Hospital, University of Champagne-Ardenne, Reims, France; ^6^Department of Biological Sciences, Immunology, UFR Odontology, University of Reims Champagne-Ardenne, Reims, France

**Keywords:** neutrophil extracellular traps, eosinophil extracellular traps, cytokine, autoimmunity, inflammation, bullous pemphigoid

## Abstract

**Background:** DNA extracellular traps (ETs), released by neutrophils (NETs), or eosinophils (EETs), play a pathogenic role in several autoimmune disorders. However, to date, NETs have never been investigated in bullous pemphigoid (BP) with respect to clinical and immunological activities, both at baseline and at time of relapse which have been characterized with specific IL-17 and IL-23 patterns.

**Objective:** We sought to assess whether ETs were associated with BP as well as the relative contribution of IL-17 axis cytokines to NET induction.

**Methods:** Skin biopsy specimens were obtained from 11 patients with BP. Immuno-detection of neutrophils and eosinophils combined to DNA staining allowed us to investigate the *in-situ* presence of NETs and EETs using confocal scanning microscopy. NETs release was evaluated *ex vivo* by stimulating polymorphonuclear cells from BP patients with BP biological fluids in presence of IL-17A and IL-23 or of glucocorticoids.

**Results:** At baseline, ETs were observed in BP lesions at the site of dermal-epidermal cleavage. Despite an important infiltrate of eosinophils, ETs were essentially associated with neutrophils *in situ* and were not related to BP clinical activity at diagnosis. *In situ* observation of NETs was associated in 6 among 8 patients with serum capacity of NET induction. Notably both blister fluid and sera from BP patients at diagnosis and at time of relapse could induce NET formation *ex vivo*. In contrast, a longitudinal investigation showed a decrease of NET formation with time of treatment in patients undergoing remission. Mimicking relapse, complementation of sera from BP patients with ongoing remission with either IL-17A or IL-23 increased NET formation. Conversely, IL-17A inhibited NET formation induced by serum from BP patients with relapse supplemented or not with IL-23. Finally, glucocorticoids also inhibited NET formation *ex vivo* in BP.

**Conclusion:** NET formation is an associated phenomenon with BP. Furthermore, we showed that IL-23 favored NET formation, whereas the effects of IL-17A are environment dependent. Indeed, IL-17A displayed a protective effect on NET formation when associated with IL-23, showing for the first-time differential effects of these two cytokines in BP.

## Introduction

Bullous pemphigoid (BP) is an invalidating autoimmune sub-epidermal blistering disease affecting preferentially the elderly. Eosinophils and neutrophils are the most represented cells in the skin inflammatory infiltrate of BP patients (1). Both cells have the capacity to form DNA traps (2–5) which play a pathogenic role in several autoimmune diseases (6, 7). The participation of eosinophil DNA extracellular traps (EET)s to blister formation has recently been reported in BP (7, 8). In contrast, neutrophil extracellular trap (NET) presence has not been demonstrated yet. Additionally, DNA trap formation has never been investigated in BP with respect to the clinical features of the disease i.e., activity and outcome. Also, the relationship between NET formation and the autoimmune or inflammatory responses, which characterize BP development, needs to be clarified.

Neutrophils and eosinophils are both involved in BP pathological process (8–13). Neutrophils play a crucial role in most BP experimental models to induce dermal-epidermal separation (10, 14–16). The cooperation with monocytic cell-line and the stimulation by CXCL-10, IL-17, or IL-23, lead neutrophils to release metalloproteinase MMP-9 and neutrophil elastase, which are responsible for dermal-epidermal junction (DEJ) disruption and BP180 cleavage (17–22). Besides, eosinophils are the predominant cells in the human BP inflammatory infiltrate (1). The activated eosinophils release a potent army of cytokines (9, 11) and granule proteins such as Eosinophil Cationic Protein (ECP), which serum concentrations parallel BP activity (23). The actions of both granulocytes converge to MMP-9 production, which suppresses the neutrophil elastase natural inhibitor, and thus contributes to dermal-epidermal splitting (24).

Stimuli inducing NETs release are numerous and highly variable. For instances auto-antibodies (AAb)s, as well as cytokines, appear to be able to induce this phenomenon (5, 6, 25). Indeed, AAbs from patients with rheumatoid arthritis and IL-17 were recently shown to induce NET formation in an *ex vivo* model of rheumatoid arthritis (25). In BP, the pathogenic role of anti-BP180 antibodies has been illustrated by both *in vitro* and *in vivo* studies, and their serum concentrations at diagnosis have been correlated with disease activity (14, 26–30). Cytokines also play a key role in BP pathogeny (17–19, 31–34). In previous studies, we showed that IL-17 levels were elevated in blister fluids, linked to a local production by neutrophils and mastocytes (17), and a relationship between IL-17 axis cytokines and BP outcome (18). More precisely, we evidenced an increased serum level of IL-23 or a high sustained serum level of IL-17 despite treatment in BP patient who later relapsed (18). Moreover, these inflammatory mediators are involved in BP pathophysiological process, as they enhance MMP-9 production by innate immune cells from patients (17, 18).

In the present study, we investigated DNA extracellular traps in BP with respect to clinical and immunological characteristics of the disease. Therefore, the aim of this study was to determine whether NETs or EETs or even both were associated with BP at tissue level, and to investigate IL-17 and IL-23 influence on NET formation *ex vivo*.

## Materials and Methods

### Patients

This prospective, observational, and translational study was conducted in our tertiary Referral Center for Autoimmune Bullous Diseases at the Reims University Hospital. Consecutive patients with newly diagnosed BP were included in this prospective study. Diagnosis of BP was made when the following criteria were met: presence of at least 3 out of 4 established clinical criteria by Vaillant et al in combination with positive direct immunofluorescence findings (30). Routine skin biopsy specimens of 11 BP patients were provided by the Pathology Department of the Reims University Hospital to perform *in situ* analysis. *Ex-vivo* assays were performed with sera from these 11 patients collected at time of diagnosis (at the same time as the biopsy) and with biological samples [sera and polymorphonuclear cells (PMN)] collected at time of diagnosis and around 150 and 360 days after, from 17 other consecutive BP patients. Sera were also collected at time of relapse in patients who underwent relapse despite treatment. PMNs used for *ex vivo* experiments were freshly isolated PMNs from patients with BP collected at any time point (between D1 and D360 after diagnosis) throughout the course of the study. Seven sera and PMN from healthy controls were provided by French Blood Agency and volunteers (mean age 66.4 years).

### *In situ* Analysis of NETs/EETs

Immunofluorescence and confocal analysis of NETs and EETs were performed on paraformaldehyde-fixed and paraffin-embedded skin biopsy specimens from 11 BP patients. DNA staining along with neutrophils and eosinophils immunostaining was performed as follow on tissues. Four consecutive deparaffanized sections were used by patient, using a distinct eosinophil marker on each. After 15 min heat retrieval in sodium citrate buffer pH6, the sections were then blocked with PBS-BSA 3% for 30 min at room temperature. Then, simultaneous staining was performed for 30 min at room temperature with mouse anti-human myeloperoxidase (1:150; *R&D Systems, Minneapolis, USA)* for neutrophil staining and another primary antibody (Ab) for eosinophil staining: either rabbit anti-human IL-5Rα (1:150; *Proteintech, Chicago, USA*), or rabbit anti-human MBP, EDN, or ECP (1:150; *Novus Biologicals, Littleton, USA*). This was followed by 30 min of incubation at room temperature with matched secondary Abs: chicken anti-rabbit IgG Alexa Fluor 488 and chicken anti-mouse IgG Alexa Fluor 594 (Invitrogen). Sytox Orange Nucleic Acid Stain (5 μM) (*Molecular Probes, Invitrogen, Carlsbad, USA)* was finally used to stain DNA prior to mounting slides with Dako® fluorescent mounting medium. The presence of NETs *in situ* was confirmed by an immunostaining performed with a rabbit polyclonal Ab to Histone H3 (1:100 overnight; *Abcam, Cambridge, UK)* revealed by a secondary chicken anti-rabbit Ab Alexa Fluor 594 (1:200. *Invitrogen, Carlsbad, USA*) and a mouse monoclonal Ab to human myeloperoxydase coupled to a FITC fluorochrome *(1:100; Abcam, Cambridge, UK)*.

Extracellular DNA filaments associated to both eosinophils and neutrophils were investigated by 2 independent investigators using 3-dimensional imaging by confocal scanning microscope LSM 710 NLO Zeiss (Zeiss SAS, Germany). Image analysis was performed by using ZEISS ZEN software (Zeiss SAS, Germany) and IMARIS Software (Bitplane, Switzerland). ETs were identified as shapes of DNA extruding from cell and stained by nucleic acid probe SYTOX orange. NETs were defined as extracellular DNA filaments stained by Sytox Orange extruding from cells labeled by MPO Ab, while cell MBP, EDN, ECP or IL-5R labeling characterized EETs.

To qualify eosinophil and neutrophil cell infiltrate, hematoxylin-eosin coloration of serial sections was performed for these 11 biopsy specimens. A rating from 0 to + + + was assigned by two operators to eosinophil and neutrophil infiltrates in the papillary dermis and in the blister cavity to allow a comparative semi-quantification of these cells.

### *Ex vivo* Analysis of NET Formation

Peripheral PMNs cells were obtained by density-gradient centrifugation from 25 mL heparinized-treated whole blood (Granulosep, Eurobio-Abcys). NET generation was performed following the protocol proposed by Brinkmann et al. (35) with the difference that SYTOX Orange was used to stain extracellular DNA. 2.10^5^ PMNs were incubated with either 5% blister fluids or 5% sera from BP patients, following preliminary optimization assays (Supplementary Figure S1). To investigate their potential influence on NET formation, recombinant IL-17A and IL-23 were added to cell culture medium at concentrations close to those determined in the sera of BP patients (1.2 ng/mL) (18). To assess the effects of treatment on NET induction by BP sera, methylprednisolone MP (10 μM), and Compound A (10 μM) were added to the cell culture medium in presence of BP sera. Each condition was tested with biological fluids from at least 3 different subjects (patients or controls).

NETs were visualized using an inverted epifluorescence microscope (AxioObserver Z1: Zeiss, Germany; camera Cool Snap HQ2: Roper Scientific, France). For each slide, a mosaic of 4 × 4 consecutive images was taken (scale image: 0.625 μm/pixel, driven by Metamorph Software (Roper scientific, Evry, France), which represents an area of around 2 mm^2^ per coverslips. Fluorescence signal was collected with bandpass 545/25 excitation filter and bandpass 605/70 emission filter. For each acquisition, fluorescence, and transmitted images were taken.

To assess NETs formation, the area occupied by the DNA filaments was determined using a dedicated home-made macro based on “skeletonize function” and the “analyse particle” tool running under IMAGE J® software (ImageJ, U. S. National Institutes of Health, Bethesda, USA.). Briefly, we skeletonized the fluorescent images, setting 40 pixels as the minimal resolution, and transformed these images into drawings of DNA filaments, which were measured and processed by the “Analyze particles” tool (Supplementary Figure S1). Area of each filament was measured, and the mean was calculated, making the conversion from pixels to μm (1pixel = 0.625 μm). The total number of cells was counted using the counter cell tool of Image J® software on the transmitted image acquired simultaneously with fluorescent image. Thus, this method allowed measuring the total number of PMNs, and the total area of NETs. Results were expressed in mean area of NETs per PMN (as proposed by Rebernick et al. (36). As variations were observed when using PMNs from different subjects, results were also expressed in ratio between mean area of NETs per cell for condition X and mean area of NETs per cell for the reference condition with the same PMNs.

### Statistical Analysis

As the distribution of the variables could not be assumed to be normal, comparisons between two groups were performed using the Wilcoxon matched pairs signed rank test for paired data and the Mann-Whitney test for unpaired data. When more of two matched groups were compared, Friedman test was performed. Ratio paired *t*-test was used to compare NETs induction by sera with and without treatment (by cytokines, methylprednisolone or compound A). Statistical difference was considered significant if *p*-value was 0.05 or less.

## Results

### DNA Extracellular Traps Mainly Originated From Neutrophils *in situ*

To investigate the presence of either NETs or EETs or even both in BP at site of lesion, immune-detection of neutrophils and eosinophils along with DNA staining was performed on paraffin-embedded skin biopsy specimens from 11 BP patients. The mean age of the 11 newly diagnosed BP patients (6 female (54.5%) and 5 males (45.5%), sex ratio F/M: 1.2) was of 78.9 years. At baseline, the clinical BP activity was assessed by the Bullous Pemphigoid Disease Assessment Index (BPDAI) score and by the number of new daily blisters. The BPDAI median value was of 53 [23–90], and 9 (81.8%) BP patients presented with a severe disease characterized by more than 10 blisters a day. Finally, 5 (45.5%) BP patients experienced a relapse despite treatment during the first year of follow up (Table 1).

**Table 1:** Individual characteristics of BP patients.

**Patient**	**NETs**		**Baseline**						**Follow-up**						
			**Age at diagnosis**	**Sex**	**Nb blisters/day**	**BPDAI**	**Serum antibodies**		**Relapse**	**Day 150**			**Day 360**		
	**Presence *in situ***	***Ex vivo* NET induction (mean area of NET μm^2^/cell)**					**Anti-BP180 (U/mL)**	**Anti-BP230 (U/mL)**		**BPDAI**	**Anti-BP180 (U/mL)**	**Anti-BP230 (U/mL)**	**BPDAI**	**Anti-BP180 (U/mL)**	**Anti-BP230 (U/mL)**
1	**+**	**1.141**	84	F	120	90^*^	**150**	**94**	**+**	34	**59**	**30**	7	**52**	**71**
2	**+**	**2.02**	95	F	30	65^*^	**64**	**83**	**+**	59^*^	**47**	7	10	**81**	**32**
3	**+**	0.285	81	F	73	65^*^	**81**	**64**	**–**	7	**22**	**14**	7	**10**	**9**
4	**+**	0.130	58	F	26	53	**56**	1	**+**	32	**74**	5	D	D	D
5	**+**	**0.471**	75	M	10	48	**116**	5	**–**	2	9	5	0	0	0
6	**+**	**0.501**	87	M	15	36	1	1	**–**	2	7	5	0	7	7
7	**+**	**0.798**	78	M	10	23	1	2	**+**	0	2	4	4	0	0
8	**+**	**0.503**	81	M	1^#^	23	9	**20**	**–**	1	7	**10**	1	7	20
9	**–**	0.382	79	M	20	73^*^	**68**	**35**	**+**	31	**41**	**21**	6	8	5
10	**–**	0.433	90	F	10	56^*^	**79**	3	**–**	5	**38**	3	10	**21**	5
11	**–**	**0.636**	60	F	5^#^	30	**26**	1	**–**	6	**16**	0	23	**11**	5

* Severe disease according to BPDAI ≥56;

*#patients with moderate disease defined by a number of new daily blisters < 10; Relapse was defined as the reappearance of at least 3 new daily blisters in between one-year follow-up. Bold characters: out of normal range values i.e., NET area per PMN >0.445 μm^2^/PMN, which is the mean value obtained by stimulation of the same PMNs with healthy sera + 2SD; Anti-BP180 Ab >9 U/ml, Anti-BP230 Ab >7 U/ml. BPDAI, bullous pemphigoid disease area index, Nb number*.

**Figure 1:**
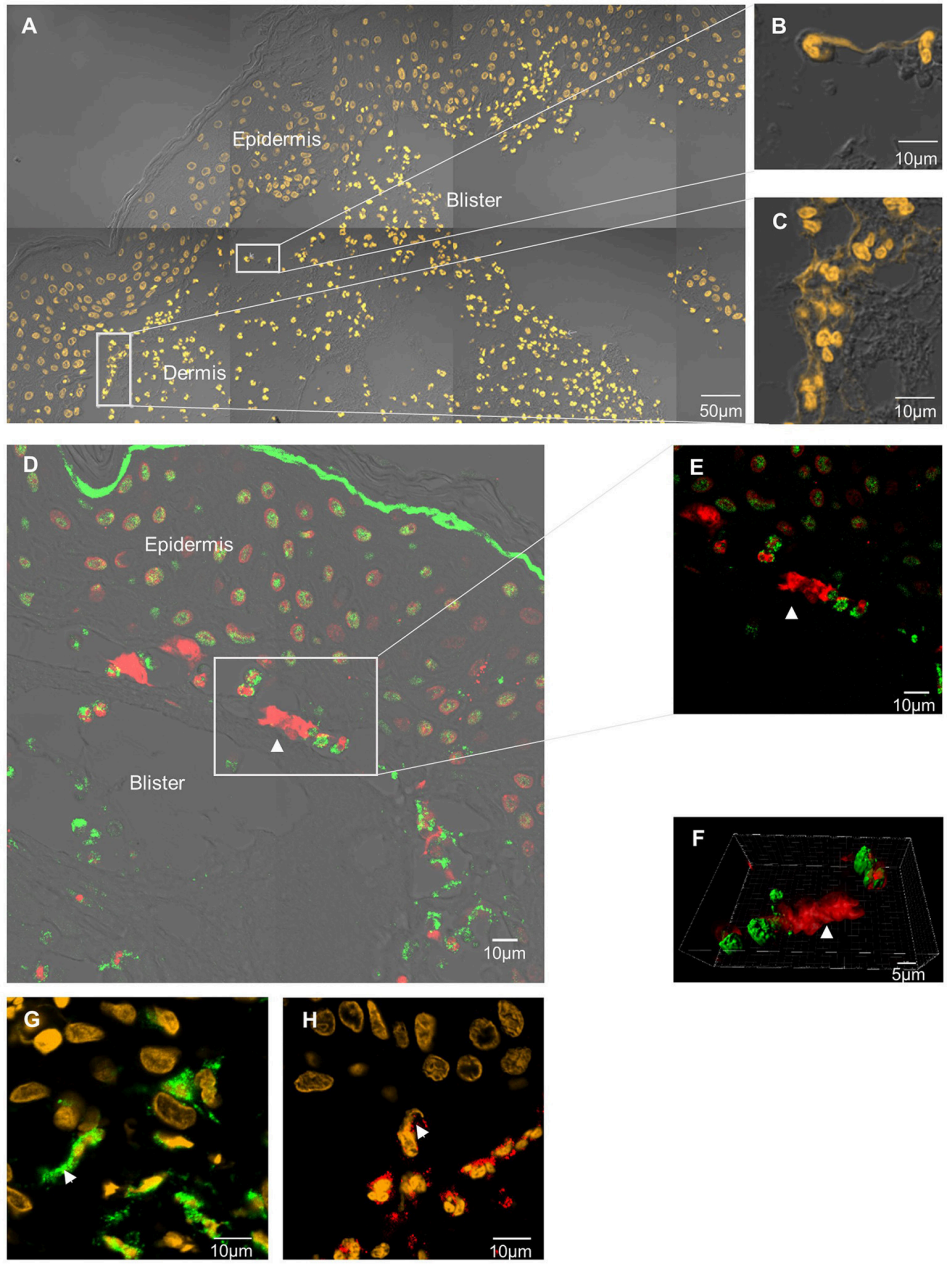
*In situ* exploration of DNA traps in BP. DNA extracellular traps are located at the edge of the DEJ splitting, in the papillary dermis of BP patient skin. Skin biopsy specimen stained by SYTOX Orange nucleic acid probe **(A–C)**. Neutrophil extracellular trap stained by anti-histone H3 Ab *(red)* and anti-myeloperoxidase Ab *(green)* **(D–F)**. 3-dimensional reconstruction imaging by confocal microscopy and IMARIS® software of this NET **(F)**. Magnification 80x, numerical aperture of 1, z-stack with 0.67μm step-size. Neutrophil extracellular trap stained by Sytox orange and anti-myeloperoxidase *(green)* **(G)**. Eosinophil extracellular trap stained by Sytox orange and anti-eosinophil-derived-neurotoxin Ab *(red)* **(H)**.

Extracellular DNA filaments were identified *in situ* in 8 out of the 11 (73%) BP patients (Figures 1A–C). NETs were observed on lesional skin biopsy specimen, in the papillary dermis at the edge of the dermal-epidermal separation (Figures 1A–G). A 3-dimensional reconstruction picture corresponding to Figure 1E allowed defining NETs as shapes of DNA extruding from neutrophils stained by MPO (Supplementary Video S1; Figure 1F). EETs were only observed in one patient despite a higher number of eosinophils than neutrophils in BP lesional skin, corroborated by both HES staining and immunofluorescence using specific eosinophils markers including MBP, EDN, ECP, or IL-5Rα (Figure 1H; Table 2). DNA extracellular trap was observed neither in blister cavity nor in perilesional dermis, despite the presence of numerous inflammatory cells. Besides, in those 8 BP patients, NETs were absent from skin biopsy specimens performed at distance of the lesions (data not shown). Also, no DNA traps were evidenced in 4 plastic surgical normal skin specimens (mean age of controls 66.5 years) (data not shown).

**Table 2:** Inflammatory cell infiltrate in BP skin according to the presence of NETs forming cells *in situ*.

**Patient**	**Blister cavity**		**Papillary dermis**		**NETS**
	**Eosinophils**	**Neutrophils**	**Eosinophils**	**Neutrophils**	
1	+	0/+	0/+	0/+	+
2	+++	+	++/+++	+/++	+
3	++	+++	0/+	0/+	+
4	++/+++	++	++/+++	++	+
5	+++	++	++	+	+
6	+++	0/+	+++	+	+
7	+++	++	++/+++	+	+
8	+	+	0/+	0/+	+
9	+++	+ /++	+++	+	–
10	+++	+/++	++	0/+	–
11	++	+++	+	+	–

*Quality of cell infiltrate assessed independently by two operators on Hematoxylin-eosin stained sections with respect to the presence of NETs determined by immunofluorescence on a serial section of the same skin biopsy. A rating from 0 to +++ was assigned to eosinophil and neutrophil infiltrates in the papillary dermis and in the blister cavity to allow a comparative semi-quantification of these cells. (n = 11)*.

*In situ* occurrence of NETs appeared to be independent of the disease activity at time of diagnosis. Indeed, patients in whom NETs were seen, showed miscellaneous BPDAI scores with values ranged from 23 to 90 and a median of 50.5 (Tables 1, 3). Furthermore, these 8 BP patients were evenly distributed according to the BPDAI score, as 3 patients displayed a BPDAI score superior to 56, 2 with a BPDAI close to mean value between 48 and 53, and 3 with a BPDAI inferior to 36. Also, NETs were observed in both patients with a multibullous BP (displaying more than 10 new blisters per day and up to 120 new daily blisters), and in those with a limited disease (down to only one blister per day). Likewise, the presence of NETs was not associated with BP relapse, as among the 8 BP patients displaying NETs at baseline, 4 later underwent a relapse and 4 remained on remission over the one-year of clinical follow-up (Table 1).

**Table 3:** Clinical and biological characteristics of BP patients according to the *in-situ* observation of NETs.

	**“NETs positive” patients**	**“NETs negative” patients**
**CLINICAL CHARACTERISTICS**		
Number of patients, *n*	8	3
Age (y); sex ratio F/M	79.9y; 1	76.3y; 2
BPDAI total score, mean ± SD	50.3 ± 23	53 ± 21.6
Total skin activity, mean ± SD	49.9 ± 23	50.6 ± 23.6
Blisters/Erosions, mean ± SD	32.2 ± 16.7	41.7 ± 13.8
Erythema/urticaria, mean ± SD	17.6 ± 9.6	9 ± 14.7
Patients with severe disease according BPDAI,^a^ *n*	3/8	2/3
Patients with at least 10 daily new blisters, *n*	7/8	2/3
Relapse^b^	4/8	1/3
**BIOLOGICAL CHARACTERISTICS**		
Positive serum anti-BP180 NC16A IgG, *n*	5/8	3/3
Mean anti BP180 NC16A IgG, U/ml	93.4	57.7
Positive serum anti-BP230 IgG, *n*	4/8	1/3

a Severe disease was defined by BPDAI ≥56.

b *Relapse was defined as the reappearance of at least 3 new daily blisters in between one-year follow-up. No statistically significant difference was observed using Mann Whitney test. n, effective, y, years. BPDAI, bullous pemphigoid disease area index*.

### Both Neutrophils and Biological Fluids From Patients With BP Were Required for NET Formation

**Figure 2:**
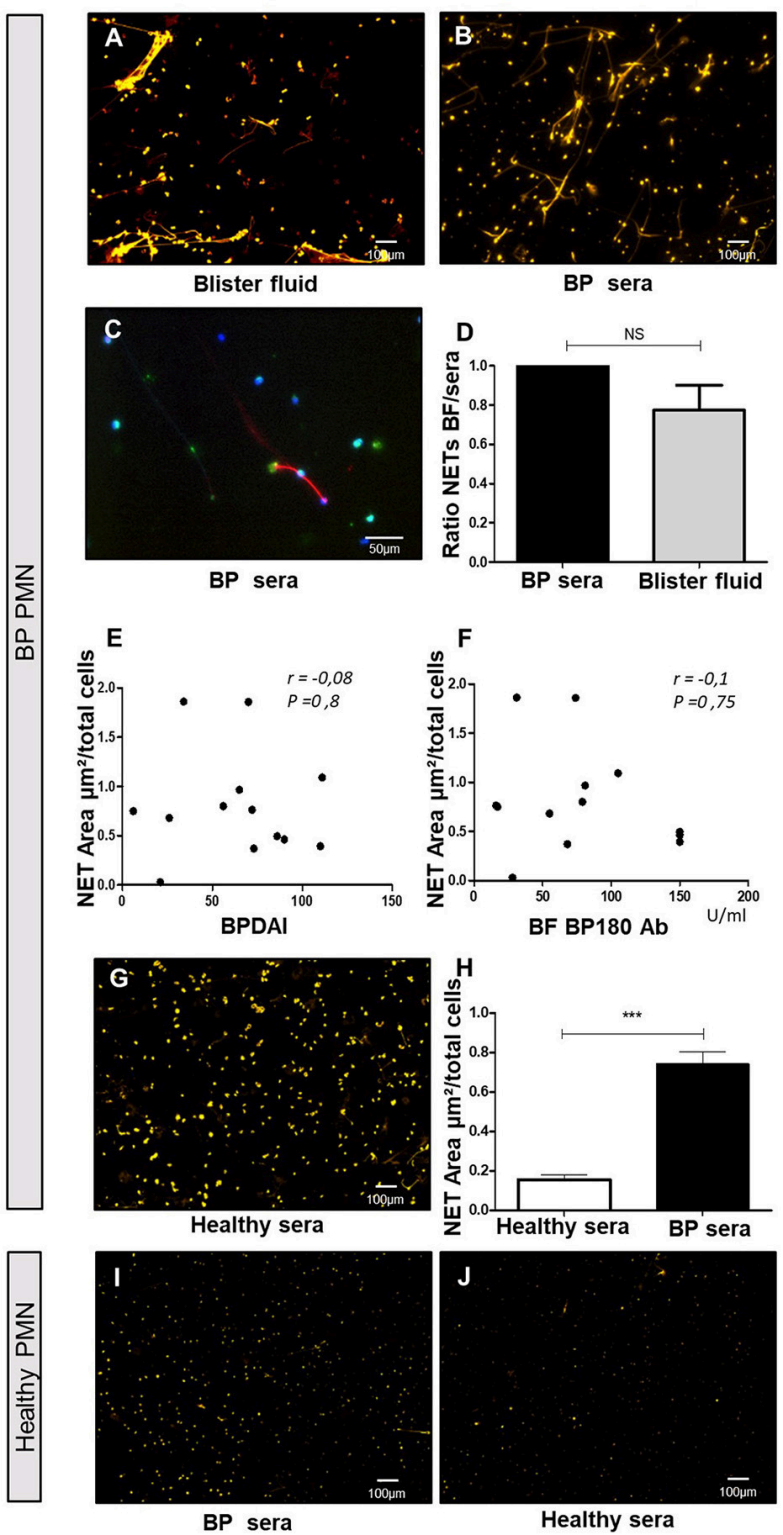
Blister fluids and BP sera induce NET formation by BP PMN but not by control PMN. *Ex vivo* stimulation of BP PMNs with BP blister fluids (BF) **(A)**, and BP sera **(B,C)** stained by Sytox orange **(A,B)** or anti-Histone H3 and anti-MPO Ab **(C)**. NET area induced by BP sera vs. BF from the same patients on heterologous BP PMNs **(D)** (*n* = 3); results expressed in ratio of NET area induced by BF to NET area induced by serum of the same patient, results represent mean ± SEM, NS, non-significant. Correlation of NET area induced by heterologous BF with BPDAI **(E)** and BF concentration of BP180 Ab **(F)**. Stimulation of BP PMN with healthy control sera **(G)** and comparison with NET area induced by BP sera **(H)** (*n* = 7), ^***^p < 0.001, results represent mean ± SEM. Stimulation of PMNs from healthy controls by BP sera **(I)**, or heterologous healthy sera **(J)**. Magnification 20x. BPDAI, Bullous Pemphigoid Disease Area Index; BF BP180 Ab, concentrations of anti-BP180 antibodies in blister fluids.

To further investigate the factors involved in NET formation associated with BP, we established an *ex vivo* NET generation model with peripheral blood PMNs from patients with BP stimulated with blister fluid collected at time of diagnosis (Figure 2A). Similar NET formation level was observed when PMNs from BP patients were stimulated with BP serum (Figures 2B–D, *p* = 0.25, Wilcoxon matched pairs test). Besides, immunostaining of myeloperoxydase and citrullinated histones allowed to confirm that neutrophils were the source of the observed NETs in this *ex vivo* model (Figure 2C). The induction of NETs by blister fluids was independent from BPDAI score at diagnosis and from BP 180 Ab levels in blister fluids (Figures 2E,F). We then investigated the capacity of the sera from the 11 BP patients for whom *in situ* exploration of NETs was performed, to induce NET formation. Among the 8 patients previously identified with NETs *in situ*, 6 showed capacity of NET induction compared with 1 patient among the 3 for whom *in-situ* assessment was negative (Table 1).

In contrast, the capability of sera from healthy donors to induce NET formation by PMN from BP patients was significantly lower than sera from BP patients (0.15 vs. 0.74 μm^2^/cell, *p* = 0.0006 Mann-Whitney test) (Figures 2G,H). To investigate whether circulating PMNs from BP patients were primed to release NETs, we assessed the ability of PMNs isolated from healthy donor peripheral blood to generate NETs upon BP serum stimulation. As showed in Figure 2I, NET quantification remained low in those conditions. Also, when PMNs from healthy donors were stimulated with heterologous sera from healthy donors, NET area remained low (Figure 2J).

### BP Serum Capacity to Induce NET Formation Decreased in BP Patients With Ongoing Remission

**Figure 3:**
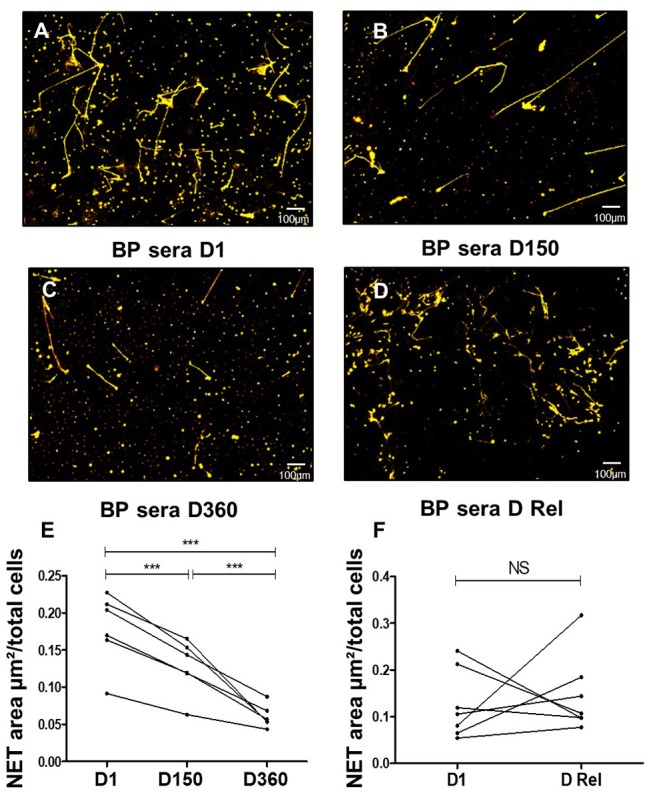
NETs are induced more efficiently by BP sera at diagnosis and at time of relapse. *Ex vivo* stimulation of BP PMNs with heterologous BP sera collected: at baseline **(A)**, around 150 days after diagnosis in patients with controlled disease **(B)**, around 360 days after diagnosis in patients undergoing remission **(C)**, or at time of relapse in patients undergoing relapse **(D)**. Magnification 20x. NET area measured for each condition: stimulation of BP PMNs with BP sera collected Baseline (D1) vs matched sera collected at Day 150 and Day 360 **(E)** (*n* = 6), ^***^p < 0.0001. NET area measured after stimulation of BP PMNs with BP sera collected at baseline (D1) vs. matched sera collected at time of relapse (D Rel) from the same patients **(F)** (*n* = 7), NS: non-significant.

Then, we evaluated the ability of BP sera collected during patient's follow up to induce NET release. Compared with baseline values, NET formation was gradually reduced with time of treatment as illustrated by *ex vivo* experiments conducted with sera collected at day150 and day 360 (0.18, 0.13, and 0.06 μm^2^/cell, *p* = 0.0001Friedman test) (Figures 3A–C,E). Conversely, NET levels remained as elevated as with BP serum at diagnosis when NET generation was performed with the BP sera collected at time of relapse from the same patients (0.13 at baseline vs. 0.15 μm^2^/cell at time of relapse, *p* = 0.68 Wilcoxon matched pairs test) (Figures 3D,F).

### IL-17 and IL-23 Display Differential Effects on NET Formation

**Figure 4:**
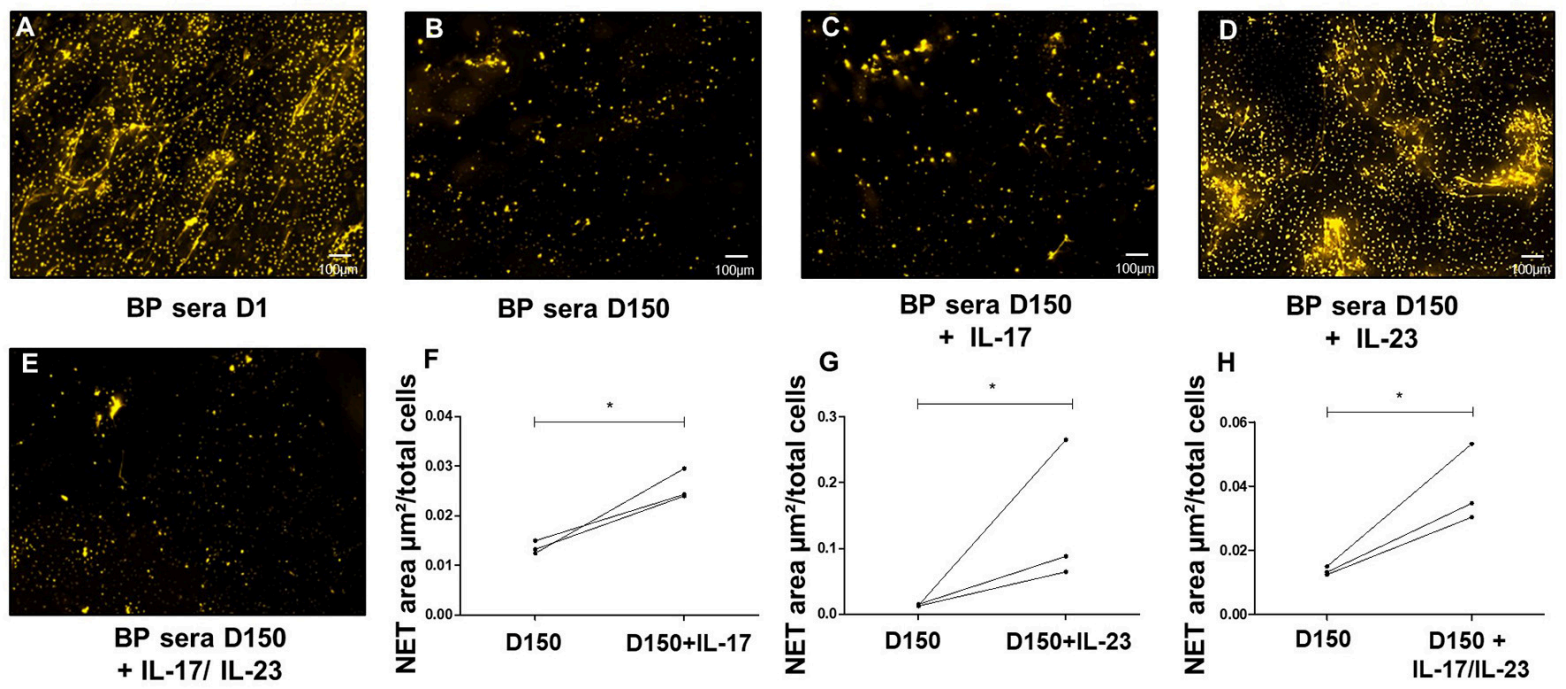
IL23 is critical to NETs formation. *Ex-vivo* stimulation of BP PMNs with BP sera collected at baseline **(A)**, around 150 days after diagnosis in patients with controlled disease **(B)**, and day 150 sera added with either IL-17A **(C)**, IL-23 **(D)**, or both IL-17A and IL-23 **(E)** (1.2 ng/mL for both cytokines). Comparison of NET area measured when cytokines IL-17A, IL-23, or both were added to day 150-serum with NET area induced by day 150- serum alone **(F–H)** (*n* = 3), ^*^ p < 0.05.

As we previously reported that IL-17 and IL-23 remained elevated or even increased in the sera of patients at risk of relapse (18), we further investigated whether these cytokines participated to NET induction in BP. In this line, we complemented BP sera collected after 150 days from patients with ongoing remission with either IL-17A or IL-23 or even both. Both IL-17A and IL-23 independently enhanced NET formation with an even greater effect under IL-23 stimulation (1.9-fold increase with IL-17, *p* = 0.029, and 10-fold increase with IL-23 with respect to the stimulation with D150 sera alone, *p* = 0.04 ratio paired *t*-test, Figures 4A–D,F,G). We then assessed the combined effects of both cytokines, IL-17A and IL-23 on NET generation. The high NET formation level induced by addition of IL-23 was reduced when IL-17A was added to the cell culture medium (Figures 4E,H).

**Figure 5:**
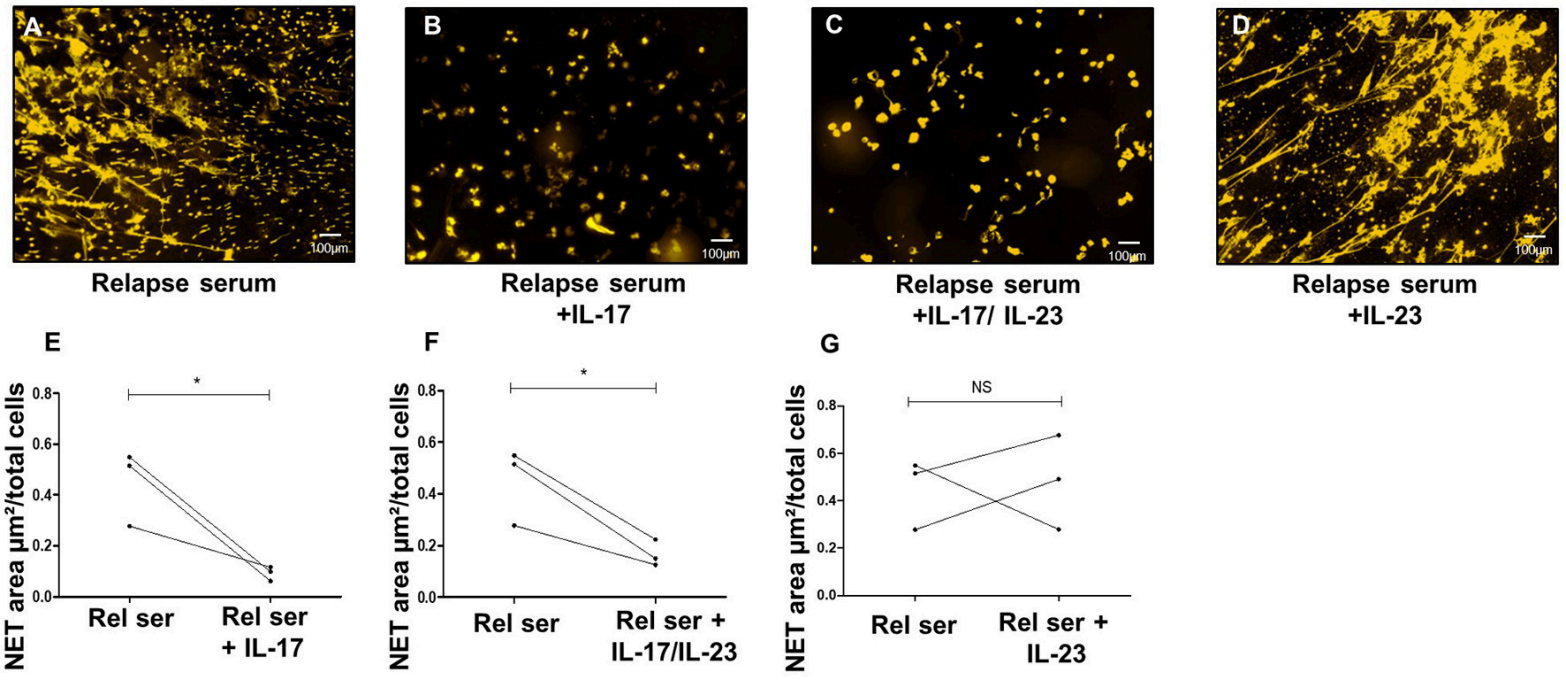
IL-17A inhibits NET formation by BP sera at time of relapse. *Ex vivo* stimulation of BP PMNs with BP sera collected at time of relapse alone **(A)**, or in presence of either IL-17 **(B)**, or both IL-17 and IL-23 **(C)**, or IL-23 **(D)**. Comparison of NET area measured when cytokines were added to serum collected at time of relapse with NET level generated in presence of this relapse serum (Rel Ser) alone **(E–G)** (*n* = 3), ^*^p < 0.05.

To further investigate the potential lowering effects of IL-17A on IL-23-induced NETs release, we added IL-17A to the potent NET inducer sera collected at the time of relapse. Supporting the above-mentioned observation, IL-17A addition (alone or combined with IL-23) significantly decreased the area occupied by NETs with respect to the area obtained with the serum of BP patients collected at time of relapse (0.09, and 0.17 vs. 0.45 μm^2^/cell, *p* = 0.05 and *p* = 0.02, respectively, ratio paired *t*-test) (Figures 5A–C,E,F). In contrast, supplementation of these sera collected at time of relapse with IL-23, did not affect their capacity to induce NET formation (0.48 vs. 0.45 μm^2^/cell, *p* = 0.89 ratio paired *t*-test) (Figures 5D,G).

### NET Formation Is Inhibited by Methylprednisolone and by Compound A

**Figure 6:**
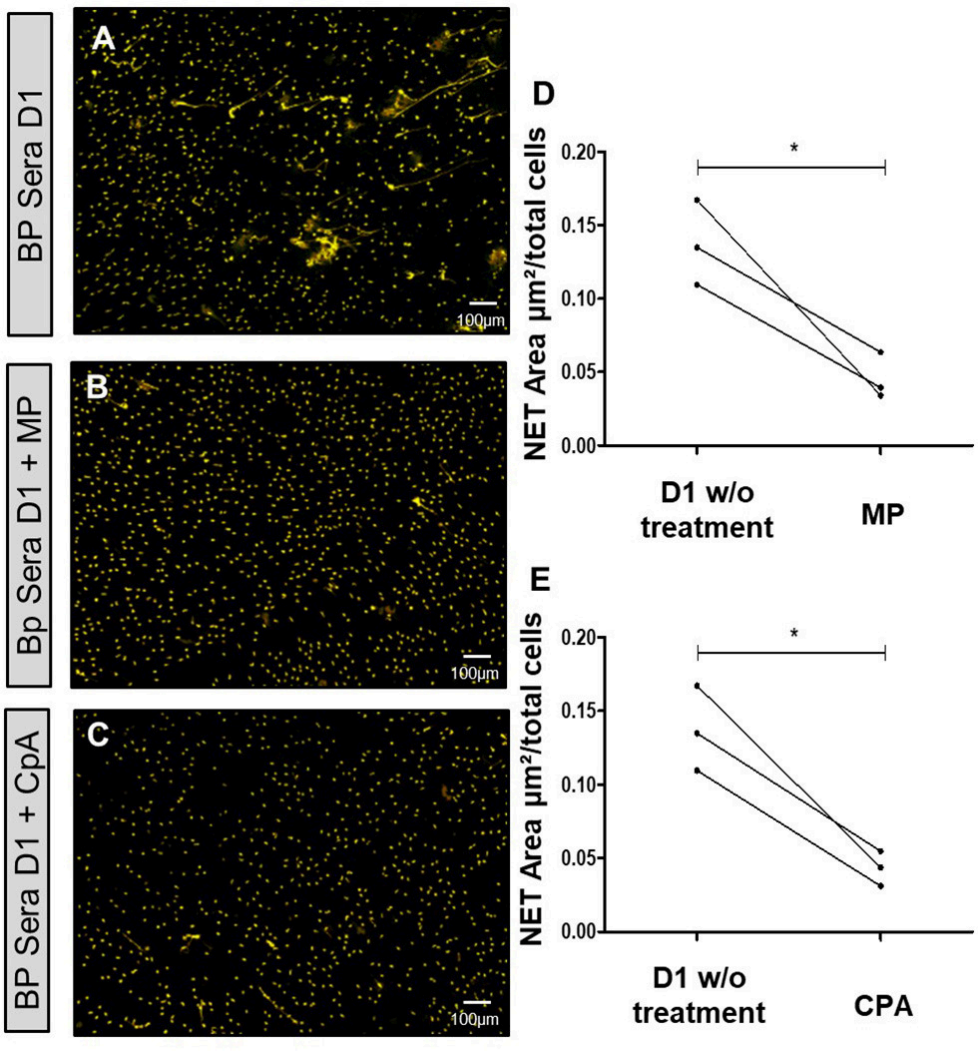
NET formation is inhibited by methylprednisolone or Compound A. *Ex vivo* stimulation of BP PMNs with BP sera collected at diagnosis (D1) alone **(A)**, or in presence of either methylprednisolone **(B)**, or compound A **(C)**. Comparison of NET area measured when treatments were added to baseline serum with NET formation by this baseline serum without treatment **(D,E)** (*n* = 3). ^*^p < 0.05, mean ± SEM are represented. MP, Methylprednisolone; CpA, Compound A; w/o, without.

Having demonstrated that the capacity of BP sera to induce NET release progressively decreased in BP patients with ongoing remission upon treatment, we then wondered whether corticosteroid therapy also reduced the capability of BP sera collected at baseline to induce NET formation. NET quantification revealed that both methylprednisolone (10 μM) and compound A (also named 2-(4-acetoxyphenyl)-2-chloro-N-ethyl ammonium chloride, a natural glucocorticoid receptor ligand (37), 10 μM) significantly decreased the capacity of BP serum to induce NET formation by BP PMNs (0.05 and 0.04 vs. 0.14 μm^2^/cell, *p* = 0.04 and *p* = 0.01, ratio paired *t*-test) (Figure 6).

## Discussion

NET formation has recently been reported in several autoimmune diseases as a phenomenon potentially associated to tissue damage and/or loss of tolerance mechanisms (6). In this study, we brought *in situ* evidence of the presence of NETs in BP at the extremity of the blister where dermis separates from epidermis. Using an *ex vivo* model, we also showed that both BP neutrophils and biological fluids (blister fluid, serum) from BP patients were required for NET formation. Analysis of NET presence at tissue level with respect to BP clinical and to immunological data revealed that NET formation was not associated with specific characteristics of the disease at baseline. However, a longitudinal analysis of NET formation using our *ex vivo* model discriminated sera from BP patients with ongoing remission under treatment from those collected at time of relapse. Based on our previous studies (17, 18), we identified IL-23 as a potent NETs enhancer in BP. In contrast, we evidenced in BP a protective role of IL-17A despite the presence of potent inducers such as IL-23 or relapse sera, demonstrating for the first time in BP antagonist functions for these 2 cytokines.

To the best of our knowledge, we here evidenced for the first time the presence *in situ* of NETs in BP. Actually, NETs were observed in 8 out of 11 patients with BP at baseline. In contrast, EETs were present only in 1 case despite the use of 4 different markers on 4 sequential sections for eosinophil immunostaining. The absence of EETs could not be explained by the quality of the inflammatory infiltrate, as eosinophils were the most represented immune cells where NETs were detected. It is possible that EETs formation rather occurs in the skin of BP patients with tissue eosinophilia as previously described (7). So far, in our study NETs were observed in the skin biopsy from BP patients whatever disease activity, although the quantity of NETs observed remained low in all cases. Thus, our observations suggest that NET formation is a common but quantitatively rare process associated with BP.

Noticeably, NETs localized precisely at the very edge of dermal-epidermal separation. NET formation could be dictated by skin microenvironment. Furthermore, most patients for whom *in situ* exploration was positive, displayed at the same time capacity to induce NET formation *ex vivo* when BP PMNs were cultured in presence of their sera. Actually, we showed that both body fluid and PMNs from BP patients were required for NET formation. Such specific NET localization at the extremity of blister where the epidermis separates from the dermis could be related to a role of NETs in blister formation as it was demonstrated using an *ex vivo* model that degradation of DNA filaments inhibited DEJ splitting (8).

Although immunoglobulins have been associated with NET formation (38–42), such process could not be foreseen in BP. Indeed, no correlation could be drawn between NETs' presence *in situ* or NET formation *ex vivo* and serum or blister fluids levels of anti-BP180 AAb. Besides, the fact that NETs were observed in most skin biopsy specimen independently of disease activity, suggests that NET inducers may be present in all body fluids from BP patients at baseline. Indeed, sera from BP patients in clinical remission were less efficient to induce NET formation. Altogether, this suggests that NET induction is rather associated with inflammatory mediators than with the autoimmune response in BP. In this setting, it was shown that inflammatory molecules such as cytokines and complement components were potent inducers of DNA traps (5, 6, 43–45).

Neutrophils' priming is a required step for NET formation in BP. In BP skin environment, the presence of cytokines able to activate innate cells such as neutrophils, eosinophils and monocytes has already been largely described (17–19, 31). Such inflammatory response may be responsible for neutrophils' priming and explain why NET formation level remained low in neutrophils from healthy donors even when stimulated with BP serum. Accordingly, it has been shown that neutrophils from normal individuals released NETs upon IL-17A stimulation only after having been primed by TNF-α (25). In BP, the importance of neutrophils was also highlighted by the capacity of both classical corticoids and Compound A to inhibit NET generation induced by serum collected at baseline. However, further studies are required to determine the cytokines involved in neutrophils sensitization to NET formation in BP.

Serum from BP patients with relapse holds on high level of NET formation. This suggests that NETs' triggers remained present in the serum of those patients even under therapy. In this line, we previously reported that the concentration of IL-17 and IL-23 remained elevated or even increased in the sera of BP patients at risk of relapse (18). Noteworthy, using our *ex vivo* model, we demonstrated that IL-23, and to a lesser extent IL-17A, could enhance NET formation in BP neutrophils. While IL-17 is known to potently recruit neutrophils, a role and the mechanisms associated with this cytokine in the induction of NET have still not been fully elucidated. Activation of p38-Mitogen-activated protein kinase (p38 MAPK) and Nuclear Factor Kappa B (NF-κB) pathways by IL-17A (46–49) may explain this property, as both are involved in NET formation (50–52), and p38 MAPK pathway mediates IL-17 induced ROS production (46). To our knowledge, the role of IL-23 in NET generation had not been evaluated yet. Neutrophils constitutively express low amount of IL-23R, which are up-regulated upon activation (53). Three activation cascades may be activated following IL-23 transduction signal among which the above-mentioned p38 MAPK and NF-κB pathways but also the mammalian target of rapamycin (mTOR) pathway which play a pivotal role in NET formation and may explain the higher capacity of IL-23 to induce NET formation in BP (50, 51, 53, 54). In this line, no correlation could be drawn between IL-17 or IL-23 blister fluids concentrations and NET formation (*r* = −0.5, *p* = 0.14, and *r* = −0.71, *p* = 0.06 respectively), suggesting that IL-17A and IL-23 were not the only triggers of NET formation in BP. Observed effects of these cytokines may also result from synergic actions of several cytokines and further studies are needed to understand mechanisms of NET induction in BP.

This study is the first to our knowledge to demonstrate tendentious functions of IL-17A with respect to IL-23 presence. Indeed, we observed a protective effect of IL-17A supplementation on NET formation induced either by IL-23 itself or by sera from BP patients with relapse, highlighting the role of disease specific microenvironment in cytokine's function. In the same line, blocking IL-17 signaling in inflammatory bowel diseases resulted in an increased expression of pro-inflammatory chemokines and cytokines (55, 56). Some molecules have recently been described in such regulation mechanisms. Suppressor of cytokine signaling 3 (SOCS3) is therefore known to inhibit Janus kinase 2 activity, thereby decreasing IL-23 induced effects (57–59). Yet, SOCS3 expression has recently been correlated with severity of inflammation, expression of proinflammatory cytokines, and activation of p38 MAPK pathway (60). SOCS3 expression is also induced by IL-17 family cytokines (61, 62). Then, although this has to be proven, we hypothesized that in BP IL-17A attenuates IL-23 induced NET formation by inducing SOCS3 expression as previously demonstrated in the airway epithelium (62). Such effects could explain the limited quantity of NETs observed in the skin of BP patients. Furthermore, our present results demonstrate that, in the future, patients with bullous pemphigoid could be further divided into subgroups according to the biomarkers expressed to identify an adequate candidate for biotherapy maintenance in relay of corticosteroid in patients at risk of relapse.

NETs are involved in loss of tolerance mechanisms in several autoimmune diseases (6). In BP, NETs release could participate to both autoimmune and inflammatory responses. Auto-antigens may be present at the surface of NETs, which prolongs their exposition, subsequently favoring an autoimmune response (63, 64). In this line, NET formation is associated with production of reactive oxygen species, and granule enzymes cover the DNA filaments (2, 6, 65). Several studies showed the involvement of NETs in tissue damage (66, 67). Brinkmann et al. reported the association of NETs with various proteases such as neutrophil elastase (5). Yet, neutrophil elastase has largely been implicated in BP pathological processes, as well as matrix metalloproteinase MMP-9 (14, 68). Thus, although NET formation does not directly correlate with BP activity, we showed in this study that NET formation depends on the presence of inflammatory cytokines, and therefore that the inflammatory response associated with BP may participate to BP antigen immunogenicity or to the perpetuation of the disease. Subsequently, our results showing NETs at the dermal-epidermal splitting area, along with the serum capacity to induce NETs at the same time, really advocate for a role of neutrophil in extracellular DNA release, although NET implication in BP still needs to be further demonstrated. Based on the literature, we hypothesize that NET-associated mechanisms may be involved in BP180 antigen immunogenicity, and further in epitope spreading in BP.

## Data Availability

All datasets generated for this study are included in the manuscript and/or the Supplementary Files.

## Ethics Statement

The investigation was conducted under the approval of the Ethic Committee of the University Hospital of Reims (CNIL authorization DR-2013-320), and all of the subjects gave their informed and written consent before participating in the study in accordance with the Helsinki Declaration.

## Author Contributions

PB, FA, and BP conceived the study. DG, EB, SL, GG, PB, FA, and BN contributed to the study design and data analysis. AD, CM, PB, and FA contributed to the clinical and histological metadata collection. DG, EB, CT, KD, SL, SN, CM, and BP contributed to the sample processing. CT conceived the macro allowing *ex vivo* NET quantification. DG, EB, and SL performed the statistical analyses. DG, PB, FA, and BP wrote the manuscript and all authors reviewed and approved the manuscript.

### Conflict of Interest Statement

The authors declare that the research was conducted in the absence of any commercial or financial relationships that could be construed as a potential conflict of interest.

## Supplementary Material

Supplementary Figure S1 Optimal culture conditions to induce NETosis with BP biological fluids. Representative microphotographs of NETs and their respective drawings generated by *ex vivo* stimulation of BP PMNs with either healthy sera **(A, A', B, B')**, BP sera **(C, C', D, D')** or BP blister fluids **(E, E', F, F')** collected at baseline. PMNs were incubated with 5% biological fluids during either 1 h **(A, A', C, C', E, E')** or 3 h **(B, B', D, D', F, F')**. NETs area generated by BP PMNs after 1 or 3 h stimulation by healthy sera, BP sera and BP BF **(G)**. BF: blister fluids.

Supplementary Video S1 3-dimensional-reconstruction of the NET corresponding to Figures 1E,F 3D-reconstruction of a netting neutrophil *in situ* by IMARIS® (Bitplane® software, Belfast, UK). Red, Histone; Green, myeloperoxidase. Magnification 80x, numerical aperture of 1, z-stack with 0.67 μm step-size.
