# Supplementary figures and images for: NET Formation in Bullous Pemphigoid Patients With Relapse Is Modulated by IL-17 and IL-23 Interplay
Source: Front Immunol. 2019 Apr 4;10:701. doi: 10.3389/fimmu.2019.00701 (PMC6458298; doi:10.3389/fimmu.2019.00701)

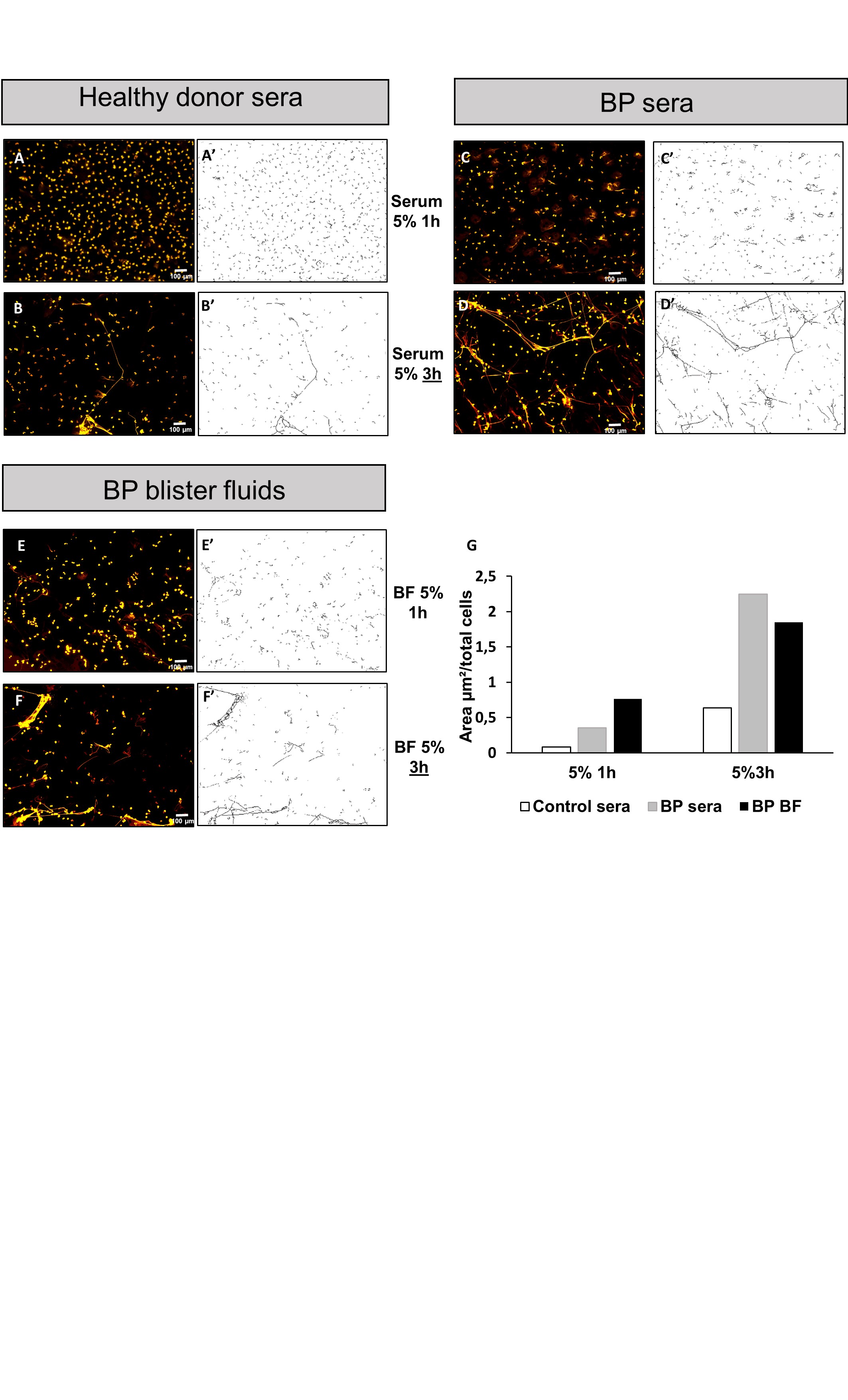

Supplement: Supplementary Figure S1 — Optimal culture conditions to induce NETosis with BP biological fluids. Representative microphotographs of NETs and their respective drawings generated by ex vivo stimulation of BP PMNs with either healthy sera (A, A', B, B'), BP sera (C, C', D, D') or BP blister fluids (E, E', F, F') collected at baseline. PMNs were incubated with 5% biological fluids during either 1 h (A, A', C, C', E, E') or 3 h (B, B', D, D', F, F'). NETs area generated by BP PMNs after 1 or 3 h stimulation by healthy sera, BP sera and BP BF (G). BF: blister fluids. [file Image_1.JPEG]
